# Utility of Three-Dimensional Holographic Workstation for Preoperative Simulation of Complex Congenital Heart Surgery

**DOI:** 10.1093/icvts/ivaf258

**Published:** 2025-10-28

**Authors:** Hiroyuki Takao, Takaya Hoashi, Taisuke Nabeshima, Takaaki Suzuki

**Affiliations:** Department of Pediatric Cardiology, International Medical Center, Saitama Medical University, Saitama 350-1298, Japan; Pediatric Cardiovascular Surgery, International Medical Center, Saitama Medical University, Saitama 350-1298, Japan; Department of Pediatric Cardiology, International Medical Center, Saitama Medical University, Saitama 350-1298, Japan; Pediatric Cardiovascular Surgery, International Medical Center, Saitama Medical University, Saitama 350-1298, Japan

**Keywords:** hologram, false Taussig-Bing anomaly, biventricular repair

## Abstract

A 7-month-old girl with false Taussig-Bing anomaly and partial atrioventricular septal defect (AVSD) was initially considered for the single-ventricle pathway. However, preoperative evaluation using a holographic workstation enabled precise ventricular volume assessment and intraventricular rerouting simulation. A virtual baffle confirmed the feasibility of biventricular repair. Volumetric analysis and cardiac magnetic resonance imaging predicted sufficient right ventricular volume post-baffle placement. The patient underwent successful biventricular repair via the Rastelli procedure and AVSD repair. Postoperative imaging verified adequate ventricular function. The holographic workstation proved cost-effective and may help determine biventricular repair viability in complex congenital heart diseases.

## INTRODUCTION

The holographic workstation has recently been applied in congenital heart surgery for imaging diagnostics.^1^ The True3D PreOP (EchoPixel, CA, USA) converts Digital Imaging and Communications in Medicine (DICOM) images from computed tomography (CT), magnetic resonance imaging (MRI), cineangiography, and echocardiography into interactive 3D holograms.^2^ Here, we report the use of this holographic workstation for preoperative surgical simulation in a case of primary biventricular repair for a false Taussig-Bing anomaly with a non-committed ventricular septal defect (VSD), pulmonary stenosis, and a partial atrioventricular septal defect (AVSD) with an intact atrial septum.

## CASE

A 7-month-old girl weighing 6.7 kg was suspected of having congenital heart disease at birth due to hypoxemia and a systolic ejection murmur. Transthoracic echocardiography showed balanced biventricular function, with both great arteries arising entirely from the right ventricle. A posteriorly positioned main pulmonary artery and a right-sided aorta confirmed a false Taussig-Bing anomaly. There was no atrioventricular valve offset, and no primum atrial septal defect. A large inlet-type VSD was noted, located far from the semilunar valves.

**Figure 1.**
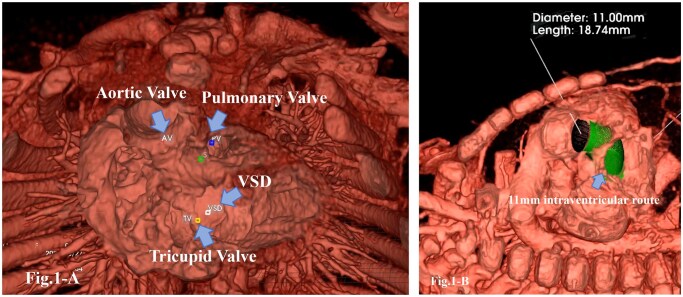
(A) Intraventricular Anatomy of Preoperative Condition of the Patient. (B) Virtual Placement of Intraventricular Route Created by Holographic Workstation (Green Portion)

Cardiac catheterization revealed a pulmonary vascular resistance of 1.1 Wood units, a pulmonary-to-systemic flow ratio of 1.18, and a pulmonary artery index (PAI) of 205 mm^2^/m^2^. High-resolution, contrast-enhanced CT DICOM data were imported into the holographic workstation to generate volumetric datasets. After segmentation and anatomical isolation, with manual editing when required, the data were reconstructed into a 3D holographic model (Fig. 1A). Using the virtual implant mode, a conduit-like left ventricular–aortic route of selected size and length was simulated for potential implantation. The aortic valve measured 11 mm in diameter, and a “virtual baffle” of the same size was placed within the model. “Virtual baffle” represents intraventricular route; LV-Aorta route (green route of Figure 1B). This simulation confirmed that the baffle would not obstruct the tricuspid valve but would partially impinge upon the pulmonary valve (Figure 1B, Video). The volume of the virtual baffle was estimated at 1.8 mL. Cardiac MRI showed a right ventricular end-diastolic volume of 12.2 mL (87% predicted; 38.6 mL/m^2^) and a left ventricular volume of 9.6 mL (65% predicted; 30.4 mL/m^2^). After subtracting the baffle volume, the effective right ventricular volume was estimated at 10.4 mL (74% predicted; 32.9 mL/m^2^), supporting the feasibility of a biventricular repair.

The patient underwent primary biventricular repair, including the Rastelli procedure and partial AVSD repair, via median sternotomy under cardiopulmonary bypass. A 15-mm right ventriculotomy was made, and an 11-mm intracardiac baffle was constructed using a 30 × 20-mm oval shaped patch fashioned from a 10-mm expanded polytetrafluoroethylene graft. The residual inlet VSD was closed with a teardrop-shaped patch. A 14-mm Contegra conduit was inserted between the branch pulmonary artery and the right ventriculotomy. The patient was weaned from bypass uneventfully. Transoesophageal echocardiography showed no pulmonary stenosis, no baffle leakage, and no pressure gradient across the left ventricle to aortic valve route.

**Figure 2.**
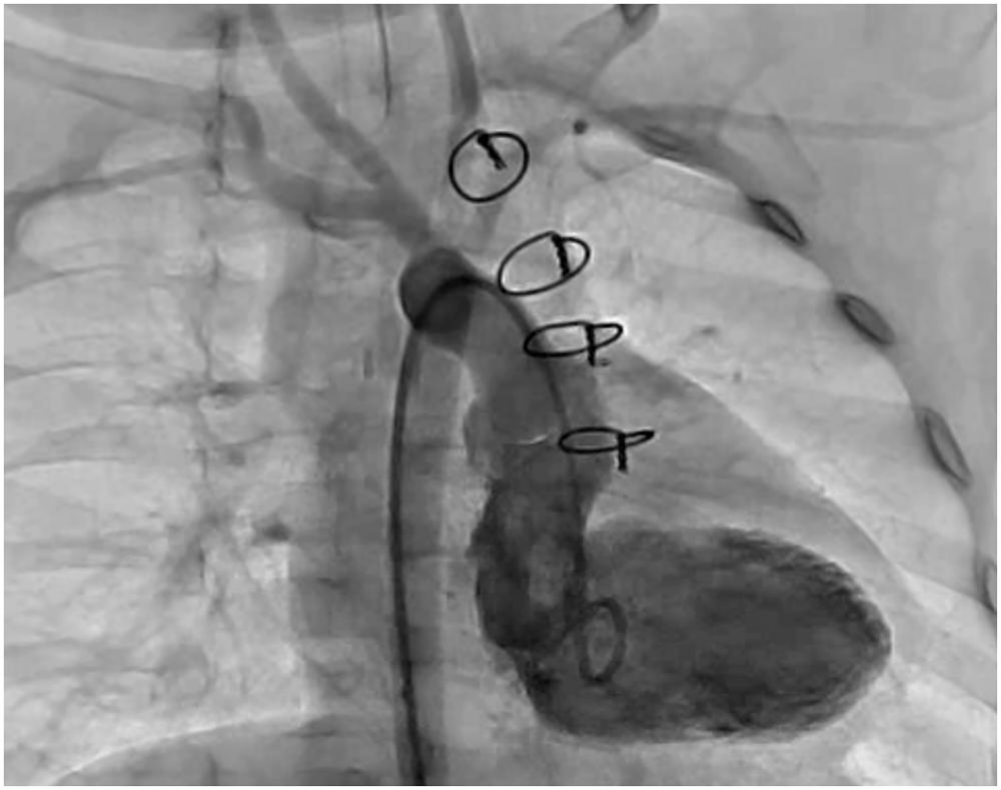
Postoperative Left Ventriculography

Postoperative left ventriculography showed that a simulated left ventricle to aortic valve route could be created (Figure 2). At 3 months, cardiac MRI revealed right and left ventricular end-diastolic volumes of 12.2 mL (76% predicted; 37.7 mL/m^2^) and 14.9 mL (88% predicted; 45.3 mL/m^2^).

**Video.**
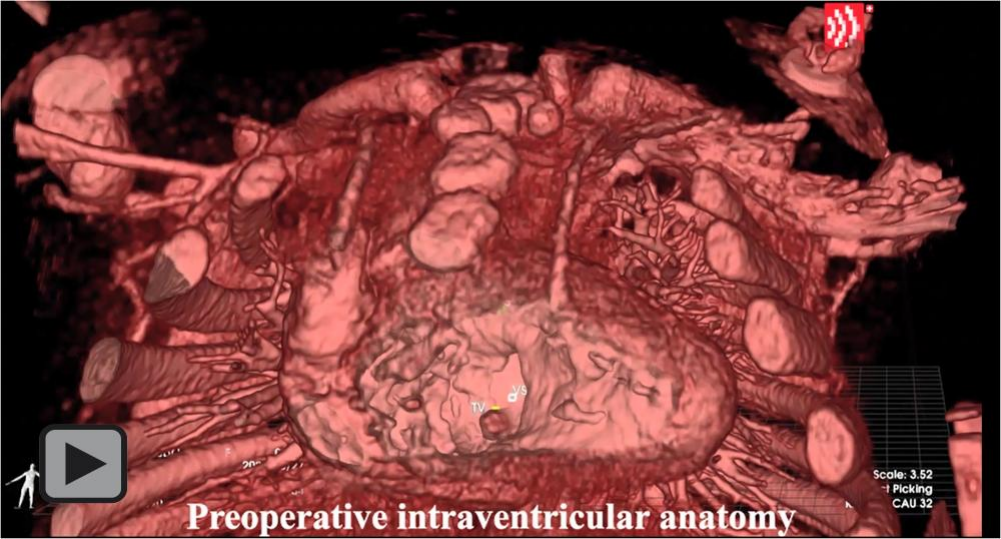
Primary Biventricular Repair for False Taussig-Bing and Partial Atrioventricular Septal Defect with Intact Atrial Septum: Preoperative Evaluation with 3D Holographic Workstation, Operative Technique, and Postoperative Angiography.

## COMMENT

In complex biventricular repair, especially as an alternative to the single ventricle pathway, careful patient selection is critical. Conditions such as double-outlet right ventricle (DORV) with remote VSD require meticulous preoperative evaluation to assess surgical feasibility.^3^

Unlike 3D-printed models, which allow for physical manipulation, simulation with hologram does not involve the actual perception of surgical techniques such as pinching, cutting, or sewing. However, holography provides a cost-effective method for immediately visualizing the spatial relationships between intracardiac and extracardiac structures, thereby facilitating surgical planning. As this was our first case, the baffle was sized intraoperatively, though patch and conduit design could theoretically be planned preoperatively with the holographic workstation.

The preoperative estimate of right ventricular volume (10.4 mL) was slightly lower than the postoperative MRI measurement (12.2 mL). This discrepancy may be due to a lack of ECG-gated imaging or the patient’s growth between assessments. Further studies are needed to validate volume assessments using holography. MRI volumetry is useful for simulating postoperative volumes in borderline cases with uncertain biventricular repair feasibility.^4^

In summary, preoperative holographic imaging with the True3D PreOP workstation enabled successful biventricular repair in a 7-month-old with DORV and AVSD. This approach helped avoid Fontan surgery and ensured sufficient ventricular volume and function, demonstrating the value of holography in complex congenital heart surgery.

## Data Availability

The data underlying this article are available from the corresponding author upon reasonable request.
